# Safety and Effectiveness of Exercise-Based Cardiac Rehabilitation for Patients With Atrial Fibrillation Following Radiofrequency Catheter Ablation Therapy: A Systematic Review and Meta-Analysis

**DOI:** 10.7759/cureus.50476

**Published:** 2023-12-13

**Authors:** Yasuyuki Kurasawa, Hirotada Maeda, Tetsuroh Tamaru, Tomohiro Sasaki, Keishi Matsumori, Yoshiharu Yokokawa, Takashi Kitagawa

**Affiliations:** 1 Division of Physical Therapy, Department of Rehabilitation, Faculty of Health Sciences, Nagano University of Health and Medicine, Nagano, JPN; 2 Department of Rehabilitation, Kanazawa Medical University Hospital, Uchinada, JPN; 3 Department of Rehabilitation, Nagano Chuo Hospital, Nagano, JPN; 4 Department of Rehabilitation, Matsumoto City Hospital, Matsumoto, JPN; 5 Department of Rehabilitation, Shinshu University Hospital, Matsumoto, JPN; 6 Department of Physical Therapy, School of Health Sciences, Shinshu University, Matsumoto, JPN

**Keywords:** systematic review, meta-analysis, radiofrequency catheter ablation, cardiac rehabilitation, atrial fibrillation

## Abstract

Despite the potential of cardiac rehabilitation (CR) to reduce atrial fibrillation (AF) recurrence after radiofrequency catheter ablation (RFCA), its prescription is not routine. We conducted a systematic review and meta-analysis to evaluate the safety and efficacy of CR in this setting. Inclusion criteria comprised randomized controlled trials (RCTs) comparing CR with usual care in patients with AF following RFCA. We performed a comprehensive search of six databases up to August 17, 2023, and conducted a thorough risk of bias assessment. We synthesized safety outcomes using AF recurrence rates to calculate relative risks (RR). Furthermore, we conducted a meta-analysis on peak oxygen uptake (VO_2_ peak) and the six-minute walk test (6MWT) to gauge efficacy, utilizing mean differences (MD) for comparison. The GRADE framework was employed to determine the certainty of evidence, with two independent reviewers completing all processes. Our analysis encompassed eight studies with 772 participants aged 55-70 years engaged in moderate-intensity CR for a median of six months. Results showed no significant difference in AF recurrence after CR (RR = 0.69 (0.41-1.14)), with low evidence certainty due to heterogeneity. Subgroup analyses suggested a poor risk reduction effect in patients with obesity and persistent AF. Significant improvements were observed in VO_2_ peak and 6MWT outcomes (VO_2_ peak; MD = 2.53 (0.78-4.28), 6MWT; MD = 38.81 (0.65-76.97)), with moderate-certainty evidence. While CR may decrease AF recurrence after RFCA, its effectiveness varies, potentially diminishing in patients with obesity or persistent AF. Moderate gains in physical performance were achieved with minimal adverse events. Further RCTs are warranted to confirm these findings.

## Introduction and background

Atrial fibrillation (AF) is the most common cardiac arrhythmia, with an estimated 2-4% prevalence among adults [1]. It accounts for the majority of hospitalizations for heart disease [2] and is associated with increased stroke incidence, mortality [3], and healthcare expenditure [4]. Patients with AF can have reduced cardiac output due to arrhythmias, resulting in diminished physical function and quality of life [5]. Moreover, AF is very closely linked to coronary artery disease (CAD), which most definitely has a bearing on cardiac events among patients with AF [6]. Therefore, the primary treatment goals for AF are to restore sinus rhythm, avoid complications, and alleviate symptoms [7]. Radiofrequency catheter ablation (RFCA) is an invasive therapy for AF that can improve physical performance and quality of life following successful treatment [8-10]. However, AF can reoccur after RFCA, and the prevention of such recurrence is crucial and has attracted much attention [11].

The prevention of AF recurrence necessitates lifestyle modifications, and cardiac rehabilitation (CR) is being highlighted as an important option [11]. CR is a multifaceted approach that includes standardized, appropriately intense exercise programs, tailored guidance, educational components, and psychological support. The intensity of CR programs can differ based on national guidelines, but they are often intended to surpass moderate levels, typically near the anaerobic threshold [12]. Furthermore, there is evidence to suggest that these programs can be beneficial for patients with CAD and valvular disease [13]. CR enhances exercise capacity and decreases complication risks in patients with AF. It also addresses obesity and hypertension, which are significant risk factors for AF recurrence and CAD [14,15]. Evidence supports the efficacy of CR in patients with AF, demonstrating improved exercise capacity without an increase in mortality or adverse events [16]. Studies have demonstrated that physical activity levels of 5-20 metabolic equivalents (METs) per week can reduce AF recurrence risk [17]. Moreover, combining weight reduction with moderate exercise levels has been found to decrease recurrent AF [18]. Thus, moderate exercise effectively improves cardiovascular health and should be offered to patients with AF after ablation, particularly those at a high risk of recurrence. However, postoperative CR is not always implemented; the nonlinear relationship between exercise and AF suggests that while moderate exercise may have a beneficial effect on AF, high-intensity exercise may induce recurrent AF [19-21]. This concern is a barrier to CR implementation.

There is no conclusive evidence that moderate-intensity CR increases the incidence of AF, and its role in AF recurrence after RFCA has not been determined. Previously, a systematic review was conducted examining the prevention of AF recurrence with exercise [22]. However, the results are uncertain because fewer than three studies were included in that review, and exercise intensity was not standardized. Another systematic review also examined CR's effect on patients after RFCA [23], but safety was not discussed, and the included papers found non-directiveness. Thus, this systematic review aimed to examine the AF recurrence associated with moderate exercise-based CR in patients with AF after RFCA, as well as with exercise function and cardiac function. We aimed to determine whether CR can be performed safely and effectively.

## Review

Materials and methods

The protocol for this systematic review is prospectively registered with protocols.io [24]. Our review and subsequent meta-analysis adhered to the Preferred Reporting Items for Systematic Reviews and Meta-Analyses (PRISMA) 2020 statement [25].

Eligibility criteria

We included randomized controlled trials (RCTs) assessing patients with AF after RFCA. Our inclusion criteria encompassed studies without limiting based on language, country, follow-up duration, or publication date, including published and unpublished studies, articles, and conference abstracts, while excluding non-RCTs. Our participant pool consisted of adults over 18 who underwent RFCA for AF, with no restrictions on sex, ethnicity, or AF type. All studies required the inclusion of CR programs with an exercise training component. We defined the intervention as a CR program, whether inpatient, outpatient, community, or home-based, that offered exercise training to improve motor skills in patients with AF. This training was expected to occur at least twice a week, lasting around one hour at moderate intensity or higher. We excluded programs without endurance training, like respiratory rehabilitation or Yoga. Studies with joint interventions, such as those including therapies in addition to rehabilitation, were included if administered equally to both experimental and control groups. Medications and nutritional therapies were considered co-interventions. The control group consisted of patients receiving usual care after RFCA without engaging in rehabilitation programs that included exercise. However, they may have received other medical advice on exercise. The primary outcome was AF recurrence, which was confirmed through an electrocardiogram (ECG), excluding the blanking period. AF recurrence was a binary variable, indicating the presence or absence of AF episodes during follow-up. Secondary outcomes included exercise capacity, measured by the six-minute walk test (6MWT) and oxygen uptake (VO_2_ peak or max), and cardiac function, assessed by left ventricular ejection fraction (LVEF). These outcomes were evaluated at baseline and post-intervention, using the nearest post-intervention measure for multi-timepoint assessments. Authors were contacted and requested to furnish additional data for under-reported outcomes of interest.

Search strategy

Our search strategy was meticulously planned and extensive, utilizing electronic databases including the Cochrane Central Register of Controlled Trials (CENTRAL), MEDLINE, Embase, Cumulative Index to Nursing and Allied Health Literature (CINAHL), Web of Science, and PEDro, executed on August 17, 2023. We modeled our participant and intervention search methodology on that of a prior Cochrane Review [16]. Utilizing key search terms like "atrial fibrillation," "catheter ablation," and "exercise," we applied the RCT filters from the Cochrane Handbook [26] to refine our search, with the complete strategy outlined in Appendix 1. To broaden our search for unpublished and ongoing studies, we searched the ClinicalTrials.gov database. We meticulously reviewed reference lists from all identified publications and relevant supplementary sources. Moreover, we examined reports for established guidelines that relate to our review topic [11,15,27-29]. The comprehensive search approach ensured the inclusion of relevant studies to support a robust and reliable systematic review and meta-analysis.

Study selection

**Figure 1:**
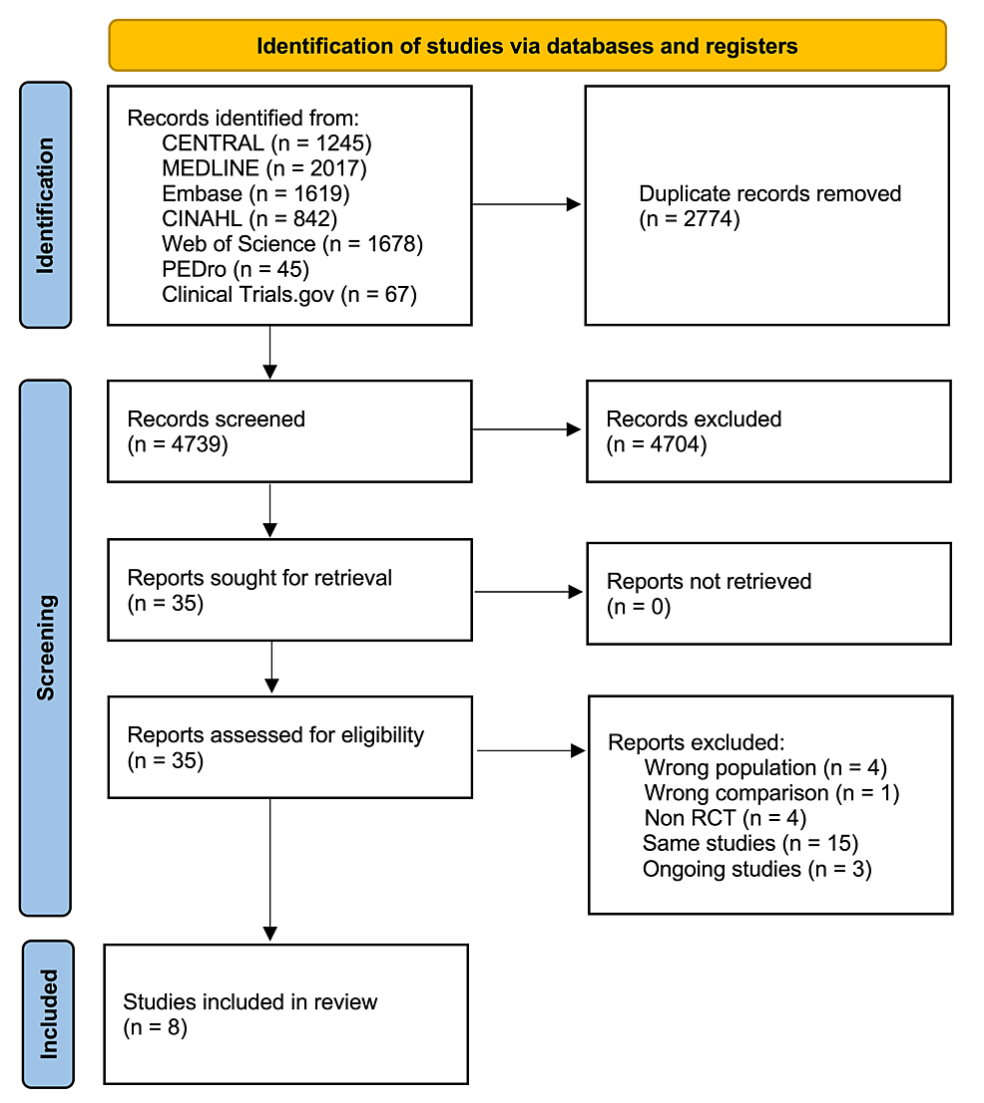
PRISMA flow diagram for an overview of the approach used to select studies

We imported all retrieved articles into Rayyan [30], a reference management software, for study selection. Duplicate reports were removed, and two independent reviewers (HM and KM) meticulously screened the titles and abstracts, excluding studies that did not meet our inclusion criteria. Subsequently, full-text versions of all potentially relevant papers were retrieved and independently assessed by the same two reviewers against the predefined inclusion criteria. Any disagreements were resolved by involving a third reviewer (YK). The study selection process is depicted using the PRISMA flow diagram, visually representing the workflow (Figure 1).

Data collection 

Two independent reviewers (HM and TT) systematically extracted and cataloged the characteristics and outcome data of the included studies using a tailored data extraction form. This form captured details such as mean age, sex, body mass index (BMI), left atrial diameter (LAD), type of AF, specifics of the intervention (type, frequency, intensity, and duration), conditions of the control group, rates of AF recurrence, measures of exercise capacity (6MWT, VO_2_ peak), and cardiac function (LVEF). Any discrepancies encountered during the data extraction process were reconciled through discussion or, when necessary, by consulting a third reviewer (YK).

Risk of bias (RoB) assessment

The risk of bias in the included studies was independently evaluated by two reviewers (HM and TS) using the Revised Cochrane Risk of Bias Tool for Randomized Trials (RoB 2) [31]. Any discrepancies in their assessments were deliberated and, if required, adjudicated by a third reviewer (YK). For each study, the risk of bias was appraised based on the following criteria: the bias emanating from the randomization process, bias due to deviations from the intended interventions, bias resulting from missing outcome data, bias in the measurement of outcomes, and bias in the selection of the reported results. We assigned the potential risk of bias as "high risk," "low risk," or "some concerns," including specific excerpts from each article with explanations for our judgments. Additionally, we compiled a summary of the RoB evaluations for all the studies.

Analyses

Given that the interventions exhibited clinical comparability regarding their nature and outcomes, we pooled the data in a meta-analysis using the Review Manager software (RevMan 5.4). The results were synthesized using a random-effects model. To evaluate treatment effects, we computed relative risk ratios (RRs) along with their 95% confidence intervals (CIs) for AF recurrence, which we treated as a dichotomous variable, and mean differences (MDs) with 95% CIs for continuous outcomes, including VO_2_ peak and LVEF. In cases where studies only provided median values, we estimated the means to normalize the data for analysis [32]. To evaluate and interpret the heterogeneity present in the included studies, we applied the I^2 ^statistic, interpreting its values with established thresholds: 0-40% for negligible heterogeneity, 30-60% for moderate heterogeneity, 50-90% for significant heterogeneity, and 75-100% for high heterogeneity. The Cochrane Q test (chi^2^ test) was also performed to assess the statistical significance of the observed heterogeneity, with a p-value of less than 0.10 considered indicative of significance [26]. To rigorously examine the effects of various factors on our findings, subgroup analyses were performed for crucial outcomes, particularly exploring the distinction between obesity (BMI of 30 or greater) and non-obesity (BMI less than 30), along with different types of AF. Studies were assigned to a specific AF category if at least 75% of their participants were diagnosed with the same AF type. In addition, sensitivity analyses were carried out, removing studies with a high risk of bias to validate the robustness and dependability of our conclusions, with subsequent comparisons of the altered results. The assessment of potential publication bias was not feasible due to the insufficient number of studies included, which was fewer than 10 [33].

Certainty of evidence (GRADE)

All outcomes were compiled in a Summary of Findings (SoF) table, and the certainty of the evidence from the review was evaluated using the GRADE methodology [34]. The RoB, inconsistency, imprecision, indirectness, and publication bias were assessed using GRADEpro GDT software for these outcomes. The Cochrane Handbook for Systematic Reviews of Interventions guided the assessment process and offered recommendations [26]. Two authors (YK and HM) independently appraised the certainty of the evidence and reached a consensus through discussion.

Results

Study selection and characteristics

Our selection process is depicted in Figure 1. We initially identified 7513 records through our search strategy. After removing 2774 duplicates, we screened 4739 titles and abstracts. From these, 35 records met the inclusion criteria and underwent full-text review. Of the 35 full-text papers assessed, 27 were excluded, resulting in the inclusion of eight studies. The details of the reviewed and excluded full-text articles are documented in Appendix 2. The final selection included five published RCTs [35-39], two conference abstracts [40,41], and one study for which authors were directly contacted to obtain data [42].

Table 1 outlines the characteristics of the eight included studies. The sample sizes ranged from 48 [38] to 210 [35], totaling 772 participants across the studies. All studies were constructed as RCTs [35-42]. Participant mean ages ranged from 55.2 to 69.9 years, with a predominance of males (52% to 100%). The types of AF included paroxysmal AF, represented in three studies [38,39,42], persistent AF in one study [36], and both paroxysmal and persistent AF in three studies [35,37,41]; one study did not specify the AF type [40]. The studies were conducted in various countries, including Denmark [35], Germany [37], Russia [38,39], China [40], Korea [41], and Japan [36,42].

**Table 1:** Characteristics of eight included studies Data are presented as mean (standard deviation) N/A: not available; AF: atrial fibrillation; BMI: body mass index; VO_2_ peak: oxygen uptake peak; 6MWT: 6-minute walk test; METs: metabolic equivalents; LVEF: left ventricular ejection fraction; LAD: left atrial diameter

Study	Region	Sample size (n)	Dropouts (%)	Age (years)	Sex (%) man	BMI (kg/m^2^)	Type of AF (%) Paroxysmal/Persistent	LVEF (%)	LAD (mm)	Outcomes	Funding
Risom et al. 2016 [35]	Denmark	210	25.2	59.5 (0.1)	74	27.5 (4.8)	72/28	N/A	N/A	AF recurrence VO_2_ peak, 6MWT	Noted
Kato M et al. 2019 [36]	Japan	68	13.2	66.0 (9.1)	80	23.9 (2.9)	0/100	65.1 (6.8)	39.4 (4.6)	AF recurrence, VO_2_ peak, 6MWT, LVEF	Noted
Kato J et al. 2019 [42]	Japan	54	13.0	69.9 (4.2)	67	24.1 (3.4)	89/11	67.5 (5.2)	37.0 (6.7)	AF recurrence, VO_2_ peak, LVEF	Noted
Baek et al. 2019 [41]	South Korea	68	N/A	56.0 (7.0)	78	N/A	51/49	N/A	N/A	AF recurrence, VO_2_ peak	N/A
Cai et al. 2019 [40]	China	56	N/A	55.2 (9.2)	79	25.5 (3.2)	N/A	N/A	N/A	VO_2 _peak, 6MWT	Noted
Gessler et al. 2021 [37]	Germany	133	0	60.3 (10.2)	62	34.9 (2.7)	42/58	58.5 (3.7)	N/A	AF recurrence, METs	Noted
Bubnova et al. 2022 [38]	Russia	48	0	56.1 (8.8)	100	28.3 (3.4)	100/0	61.9 (6.5)	44.6 (3.9)	AF recurrence, LVEF	Noted
Pogosova et al. 2023 [39]	Russia	135	0	57.3 (9.1)	52	29.8 (4.2)	100/0	N/A	N/A	AF recurrence	Noted

Exercise interventions

Table 2 details the exercise interventions employed across the eight studies included in our review. The median duration for the exercise programs was six months, ranging from a minimum of two months [40] to a maximum of twelve months [39,41]. The interventions primarily focused on moderate-intensity or higher CR, including endurance and resistance training exercises. Most programs were facility-based, offering supervised CR sessions and guidance for home exercises. Two studies diverged by offering home-based remote CR [39,40], enhancing accessibility and potentially improving adherence through convenience. Some programs were enriched with non-exercise components, including psychoeducation, [35], weight management [37], and behavioral modification strategies [40], contributing to a holistic CR approach. The frequency of the interventions varied, with four studies conducting sessions 1-3 times per week, two studies providing sessions at least once a month [37,41], and the remaining two studies not specifying the frequency [39,40]. The intensity of exercises was anchored at a moderate level, corresponding to 13-15 on the Borg Scale of Perceived Exertion and 40-60% of the repetition maximum for resistance exercises. The duration of these sessions typically spanned from 30 to 60 minutes, with this aspect not reported in two studies [39,41]. The control conditions were typically usual care or follow-up, except for one study [42] that incorporated a short-term, one-month CR intervention. This spectrum of intervention strategies showcases the variety of CR programs implemented across the different studies, offering a breadth of data on the effects of CR post-RFCA for patients with AF.

**Table 2:** Characteristics of the exercise program included in eight studies N/A: not available; AT: anaerobic threshold; RM: repetition maximum

Study	Follow-up (month)	Type of exercise	Frequency (times/week)	Intensity	Time of exercise (minutes)	Other programs	Control
Risom et al. 2016 [35]	3	Endurance and resistance training	3	Borg scale 15	60	Psychoeducational consultations	Standard follow-up
Kato et al. 2019 [36]	6	Endurance and resistance training, and walking exercise	3–5	Endurance training: AT lord. Resistance training 40–60%1RM	30–60	N/A	Usual care
Kato et al. 2019 [42]	5	Endurance and resistance training, and walking exercise	1–3	AT lord or Borg scale 13	45	N/A	1-month training
Baek et al. 2019 [41]	12	Aerobic interval training	18	Aerobic training range	N/A	N/A	Usual care
Cai et al. 2019 [40]	2	Home-based physical exercise	N/A	Target heart rate exercise	150/week	Smartphone-based follow-up	Usual care
Gessler et al. 2021 [37]	6	Endurance and resistance, and aqua training	8 sessions	Moderate	60×2	Medical supervision, dietary guidance, cognitive behavior therapy for weight loss	Usual care
Bubnova et al. 2022 [38]	6	Comprehensive exercise	3 (minimum)	Moderate	45	N/A	Standard follow-up
Pogosova et al. 2023 [39]	12	Home-based physical exercise	N/A	Moderate to vigorous physical activity	N/A	Individual counseling, remote support by phone and email	Usual care

RoB

**Figure 2:**
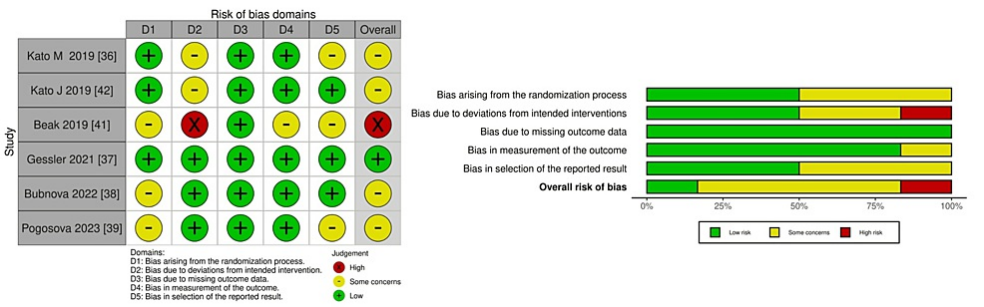
Risk of bias summary and graph for atrial fibrillation (AF) recurrence

**Figure 3:**
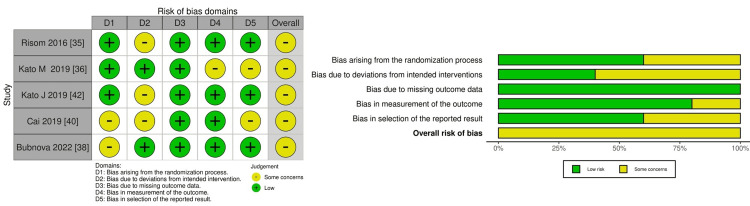
Risk of bias summary and graph for exercise capacity

**Figure 4:**
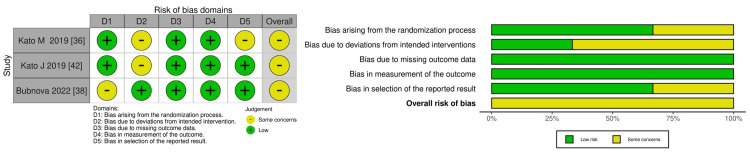
Risk of bias summary and graph for left ventricular ejection fraction (LVEF)

The RoB results, as visualized using the Robvis (visualization tool) [43], for each outcome are shown in Figures 2-4. Each outcome was meticulously evaluated for potential biases. For AF recurrence, the overall RoB was classified as "some concerns" to "high." Several factors influenced this categorization, including the process of randomization, deviations from the planned intervention, and the reporting of the results (Figure 2).

When examining exercise capacity, we also assigned an overall RoB of "some concerns." This paralleled the AF recurrence assessment, with additional concerns arising from missing outcome data. Specifically, the 6MWT results may have been influenced by patients' awareness of the intervention, which contributed to the RoB classification, as illustrated in Figure 3.

Lastly, the LVEF outcome was similarly rated as having "some concerns" regarding RoB. Here, the concerns were primarily related to deviations from the intervention, the completeness of the outcome data, and the choice of reported results, as presented in Figure 4.

The consistent "some concerns" designation across various aspects necessitated a cautious interpretation of the findings, recognizing the potential biases influencing the study outcomes.

Effect of CR on patients with RFCA

The meta-analysis assessed the impact of CR on patients with AF who underwent RFCA, focusing on AF recurrence, exercise capacity, and LVEF. We conducted an exhaustive analysis, including conference abstracts and unpublished papers. Subsequently, these sources were excluded from the sensitivity analysis to confirm the robustness of the results.

**Figure 5:**
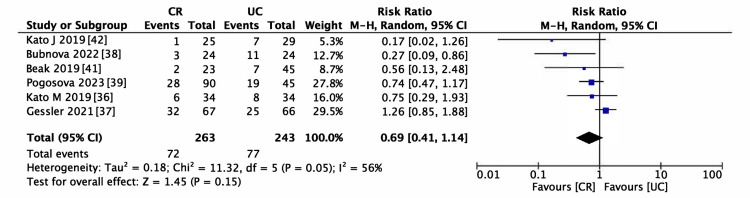
Forest plot for atrial fibrillation (AF) recurrence CR: cardiac rehabilitation; UC: usual care

**Figure 6:**
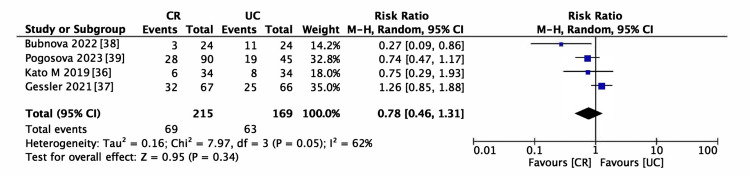
Forest plot for sensitivity analysis of atrial fibrillation (AF) recurrence CR: cardiac rehabilitation; UC: usual care

**Figure 7:**
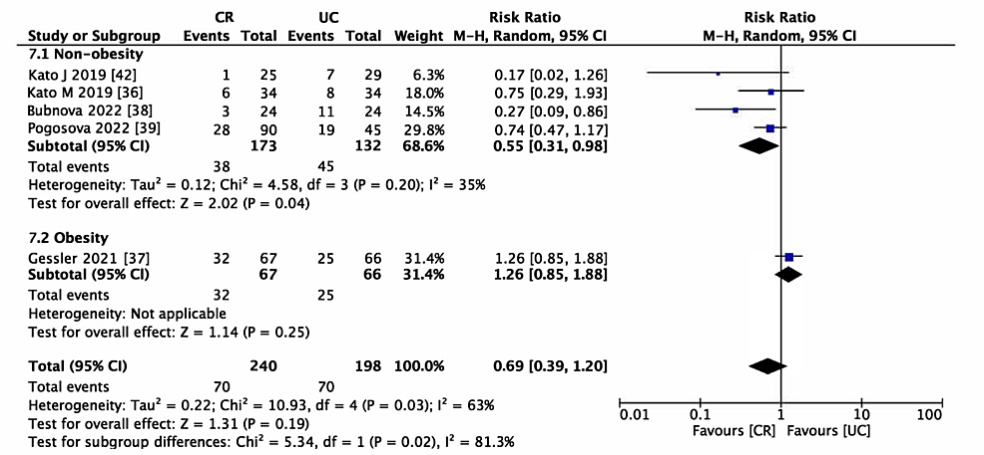
Forest plot of atrial fibrillation (AF) recurrence (obese vs. non-obese subgroup analysis) CR: cardiac rehabilitation; UC: usual care

**Figure 8:**
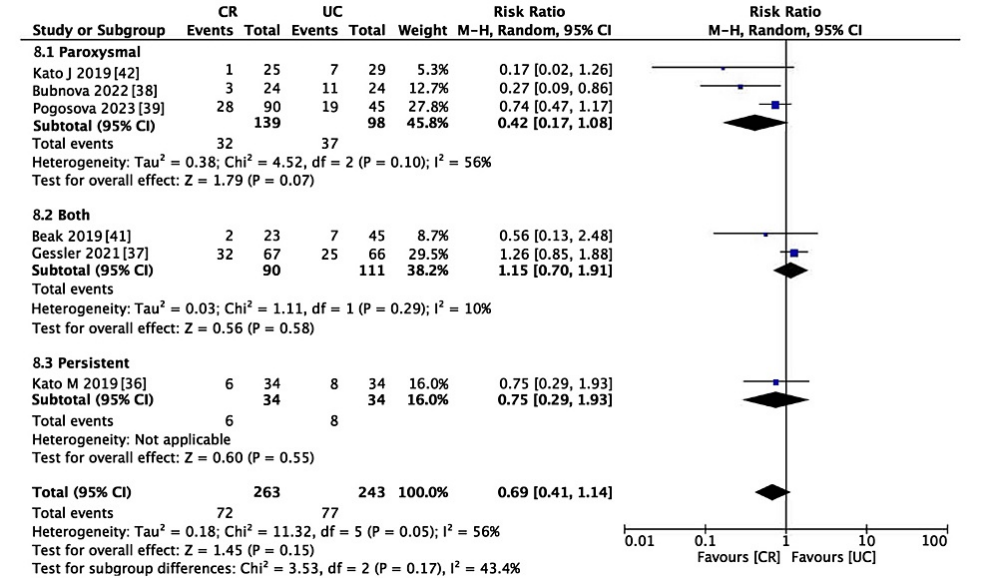
Forest plot of atrial fibrillation (AF) recurrence (AF type subgroup analysis) CR: cardiac rehabilitation; UC: usual care

Six studies encompassing 506 participants met the inclusion criteria for evaluating AF recurrence [36-39,41,42]. The incidence of AF recurrence was lower in the CR group (RR = 0.69, 95% CI (0.41-1.14), p = 0.15; Figure 5); however, the reduction was not statistically significant. The heterogeneity among the studies was moderate (I^2^ > 50%). This pattern persisted even in the sensitivity analysis, as depicted in Figure 6 (RR = 0.78, 95% CI (0.46-1.31)). Consequently, subgroup analyses were performed to identify potential sources of heterogeneity. Within the BMI categories (Figure 7), a statistically significant reduction in the risk of AF recurrence was noted for the non-obesity subgroup (RR = 0.55, 95% CI (0.31-0.98)). In contrast, the obesity subgroup did not show a significant reduction in the risk of AF recurrence. Significant differences between subgroups were observed, partially explaining the heterogeneity. Different types of AF were also examined; the paroxysmal AF subgroup showed a slightly reduced risk of recurrence, although statistical significance was not attained. Furthermore, there was a smaller effect size in risk reduction in subgroups consisting solely of or including persistent AF (Figure 8). Despite these observations, the subgroup analysis did not fully resolve the heterogeneity issue. Moreover, the limited number of studies could have resulted in underpowered statistical conclusions. 

**Figure 9:**
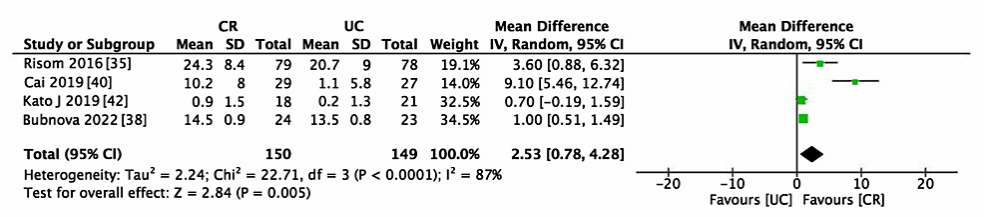
Forest plot for oxygen uptake (VO2) peak CR: cardiac rehabilitation; UC: usual care.

**Figure 10:**
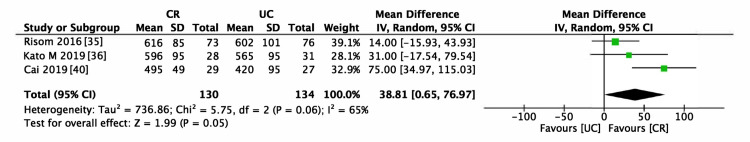
Forest plot for six-minute walk test (6MWT) CR: cardiac rehabilitation; UC: usual care

The meta-analysis incorporated five studies evaluating exercise capacity, specifically VO_2_ peak [35,38,40,42] and 6MWT [35,36,40], with 299 and 264 participants respectively. We intended to incorporate these results into standard mean difference calculations. However, due to a combination of post-intervention results and change scores, we computed separate mean differences for each measure (6MWT and VO_2_ peak). Additionally, one RCT [35], lacked sufficient data for analysis; thus, we used values from an earlier systematic review [16] that had analyzed the same RCT. The VO_2_ peak significantly increased in the CR group (MD = 2.53 ml/kg/min (95% CI 0.78-4.28), p = 0.07; Figure 9). Similarly, the 6MWT scores significantly improved in the CR group (MD = 38.81 m (95% CI 0.65-76.97), p = 0.05; Figure 10). Both outcomes exhibited moderate heterogeneity (I^2 ^> 50%), as presented in Figure 9 and Figure 10. However, due to the limited number of studies, subgroup analyses were not feasible.

**Figure 11:**
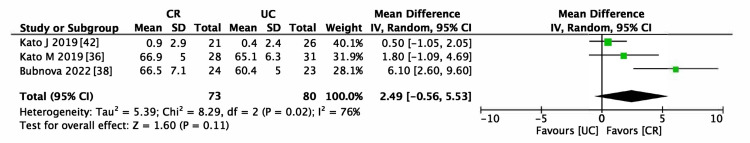
Forest plot for left ventricular ejection fraction (LVEF) CR: cardiac rehabilitation; UC: usual care

In the analysis of LVEF, three studies, with a total of 153 participants, were included [36,38,42]. LVEF did not exhibit improvement in the CR group (MD = 2.49, 95% CI (-0.56-5.53), p = 0.26; Figure 11).

Grading of evidence

Table 3 presents the certainty of the evidence in the SoF table format. We determined that there needs to be more certainty in the evidence regarding the safety of CR in patients with AF after RFCA. The evidence for AF recurrence was deemed low, exhibiting heterogeneity in the pooled results (Figure 5). Obesity and the type of AF were identified as potential effect modifiers. Additionally, the optimal information size required for a definitive conclusion had not been attained, necessitating a further downgrade in the evidence level. The risk of bias was not downgraded due to the low contribution of studies that presented "high." The evidence for the effectiveness of CR in improving motor performance, as assessed by the 6MWT and VO_2_ peak, was regarded as moderate. It was downgraded due to observed heterogeneity (Figure 9 and Figure 10). Although the effects of CR on these measures were statistically significant, the observed variability in the potential for small study effects indicates a likelihood of overestimated effects in smaller studies compared to larger ones. The certainty of the evidence regarding cardiac function, evaluated by LVEF, was low. This assessment was influenced by the heterogeneity illustrated in Figure 11 and the insufficient data quantity to establish robust conclusions.

**Table 3:** Summary of findings table in this review *The risk in the intervention group (and its 95% confidence interval) is based on the assumed risk in the comparison group and the relative effect of the intervention (and its 95% CI). CI: confidence interval; MD: mean difference; RR: risk ratio; AF: atrial fibrillation; VO_2_ peak: oxygen uptake peak; 6MWT: six-minute walk test; LVEF: left ventricular ejection fraction. *GRADE Working Group grades of evidence* High certainty: we are very confident that the true effect lies close to that of the estimate of the effect. Moderate certainty: we are moderately confident in the effect estimate: the true effect is likely to be close to the estimate of the effect, but there is a possibility that it is substantially different. Low certainty: our confidence in the effect estimate is limited: the true effect may be substantially different from the estimate of the effect. Very low certainty: we have very little confidence in the effect estimate: the true effect is likely to be substantially different from the estimate of effect. *Explanations* ^a^Downgraded one level for clinical inconsistencies of different AF recurrence risks, such as type of AF and body mass index, were a concern. ^b^Downgraded one level for concerns about imprecision falling short of the requisite optimal information size, and the 95% CI encompassed a range that included no effect but not a harmful range (95% CI = 0.41–1.14). ^c^Downgraded one level for a potential small study effect was suspected. ^d^Downgraded one level for concerns about imprecision falling short of the requisite optimal information size, and the 95% CI encompassed a range that included no effect but not harmful range (95% CI = -0.56=5.53).

Outcomes	Anticipated absolute effects^*^ (95% CI)		Relative effect (95% CI)	No of participants (studies)	Certainty of the evidence (GRADE)
							Risk with usual care	Risk with cardiac rehabilitation			
AF recurrence	317 per 1,000	219 per 1,000 (130 to 361)	RR 0.69 (0.41 to 1.14)	506 (6 RCTs)	⨁⨁◯◯
					Low^a,b^
VO_2_peak	The mean VO_2 _peak was 0	MD 2.53 higher (0.78 higher to 4.28 higher)	-	299 (4 RCTs)	⨁⨁⨁◯
					Moderate^c^
6MWT	The mean 6MWT was 0	MD 38.81 higher (0.65 higher to 76.97 higher)	-	264 (3 RCTs)	⨁⨁⨁◯
					Moderate^c^
LVEF	The mean LVEF was 0	MD 2.49 higher (0.56 lower to 5.53 higher)	-	153(3 RCTs)	⨁⨁◯◯
					Low^c,d^

Discussion

This systematic review and meta-analysis have yielded several crucial insights. CR might not have any effect on or even decrease the risk of AF recurrence. However, the level of certainty in this evidence is not robust. Our subgroup analysis, exploring the heterogeneity in factors such as BMI and the type of AF, suggests that the favorable effects of CR may be less pronounced in individuals with obesity and in those with persistent AF. Additionally, CR appears to impact exercise capacity positively, and the evidence supporting this outcome is of moderate certainty. Regarding LVEF, the impact of CR ranges from negligible to none, but the evidence backing this finding is of low certainty.

This review demonstrated that incorporating CR for patients with AF following RFCA either diminished or did not alter the risk of AF recurrence. This finding stands in contrast to prior systematic reviews [22]. The preceding review reported a reduction in AF recurrence; however, only three studies were included in the meta-analysis that examined AF recurrence. The variation in findings could be due to the two effect modifiers identified in our subgroup analysis, BMI and the type of AF, which seem to influence the outcomes.

Concerning BMI, obesity has been identified as an independent risk factor for the onset of AF. This relationship persists for both AF recurrence and AF burden, with an estimated increase in the incidence of AF for each increase in BMI [44]. Within our analysis, one study focused on obese individuals, defined by a BMI of 30 or greater. The study also noted that weight loss was inadequate, and notably, it singularly indicated an augmented risk of developing the condition. Participants in this study likely faced a higher recurrence risk than participants in other studies, introducing potential heterogeneity and diminishing the overall efficacy of the interventions. Given that obesity is a modifiable risk factor, lifestyle interventions, including dietary changes and physical activity, should ideally mitigate risk factors. Such interventions could concurrently address related comorbid risk factors like hypertension, sleep apnea, and impaired glucose regulation [45]. A comprehensive CR approach incorporating exercise, education, and lifestyle changes, might effectively tackle these issues [14].

In terms of the type of AF, several studies have considered the type of AF as a prognostic indicator for recurrence [46-49]. Our analysis aligns with this perspective, indicating that studies predominantly involving paroxysmal AF exhibited a more pronounced risk reduction. In contrast, those with persistent AF, or a more significant proportion thereof, demonstrated a lesser effect. It has been posited that patients enduring AF for protracted durations, accompanied by electrical and structural remodeling, exhibit a heightened risk of recurrent AF, potentially diminishing the prophylactic impact of CR. However, as AF recurrence depends on individual patient factors such as age, sex, and other complications, the aforementioned studies may have included patients at higher risk for such recurrence.

The limited number of studies included in our analysis hindered a thorough examination of additional factors; thus, fully resolving heterogeneity remains a challenge. Nevertheless, the findings suggest that moderate-intensity CR not only may not induce AF recurrence but also potentially decreases the risk. Given that CR may not uniformly benefit patients at a high risk of recurrence, such as those with obesity, its implementation should be approached with prospective safety considerations.

The impact of CR on exercise performance following RFCA appears promising, potentially enhancing physical capabilities. This is supported by evidence of moderate certainty, indicated by increased VO_2_ peak and improvements in the 6MWT outcomes, consistent with findings from prior systematic reviews [23]. The improvement in VO_2_ peak observed in our study significantly exceeds the minimum clinically significant difference (MCID) of 1 mL/kg [50], with an MD of 2.53 mL/kg. Furthermore, for the 6MWT, the MCID for patients with various conditions, including cardiac diseases, is estimated to be between 14.0 and 30.5 meters [51]. Our results surpass this threshold, showing an MD of 38.81 meters. Thus, CR demonstrates a notable efficacy in enhancing physical performance in patients after RFCA, and it should be considered as a means to improve physical fitness in these patients. However, the provision of outpatient CR is limited, often due to resource constraints and patient factors. Therefore, further attention and focus on innovative virtual and remote CR programs is warranted [52]. One RCT comparing post-RFCA hospital-based supervised CR with remote CR facilitated by a mobile application revealed results comparable to those of traditional hospital-based programs, indicating that not only the physical activity and exercise capacity improved, but also patient self-efficacy increased [53]. Therefore, it is imperative to offer adaptable and effective rehabilitation strategies tailored to each patient's individual needs and situations.

As for the effect of CR on patient cardiac function after RCFA, CR may not affect LVEF. This result was contrary to a previous systematic review [23], which revealed a small effect, with an MD of 0.09 (95%CI 0.01, 0.17). While other reports have indicated improved LVEF in patients with heart failure following CR, the studies included in our analysis [36,38,42] reported normal baseline LVEF values. This suggests that the LVEF in these patients might have already been enhanced due to the RFCA [54]. Consequently, CR may have a limited impact on improving LVEF in patients undergoing RFCA. However, this result is uncertain due to lack of information in the included studies.

In this systematic review, RCTs were rigorously selected to focus exclusively on patients after RFCA, with exercise intensity standardized at a moderate level. Notably, this is the first systematic review to specifically investigate the impact of CR on reducing AF recurrence following RFCA. However, this study had several limitations. First, while we addressed some aspects of heterogeneity, the RFCA patient population is inherently diverse. This population includes individuals with varying demographic factors such as age and gender, as well as a range of risk factors like heart disease, diabetes, hypertension, and hyperlipidemia ongoing drug therapy. Such diversity potentially contributes to the heterogeneity observed both within and across studies. We were unable to resolve the presence of such heterogeneity. In addition, the presence of CAD has recently been noted as a risk factor for recurrence after RCFA, but this could not be investigated [55]. Second, our analysis compared the most recent data on AF recurrence at the end of the intervention period, which ranged from 2 to 12 months. This variability in the CR provision period might have contributed to additional heterogeneity across studies. Furthermore, this review did not evaluate the long-term effects, extending beyond one year. Consequently, the ability to detect event occurrences might be limited, highlighting the need for more comprehensive long-term follow-up studies. Lastly, the methodologies used to determine AF recurrence varied across the included studies, ranging from 24-hour Holter ECG monitoring to implantable loop recorders. Additionally, some studies needed more detailed specifications regarding their methods for detecting recurrence. Thus, the detection power of event occurrence may be low. For AF recurrence to be effectively evaluated as an outcome, there is a need for more precise assessment techniques and thorough documentation of the employed methodologies.

## Conclusions

This systematic review and meta-analysis indicate that CR following RFCA for AF may either reduce or have no impact on AF recurrence, suggesting its safety. However, the effectiveness of CR could be diminished in patients with obesity or other factors that increase the risk of AF recurrence. Thus, a tailored, individualized approach to CR should be considered, especially for those at a high risk of recurrence. Regarding physical performance, the evidence demonstrates moderate efficacy in improvement, supporting the potential benefits of CR in this aspect. While further RCTs are necessary to establish more conclusive evidence, the current findings suggest a low risk of adverse effects associated with CR. These results reinforce the notion of pursuing CR for patients after RFCA, highlighting its potential benefits and relative safety.

## Appendices

**Table 4:** Search strategies for systematic review

CENTRAL (via Ovid) search strategy	
Participant Keywords:	
1	exp Atrial Fibrillation/
2	atrial fibrillation*.tw.
3	auricular fibrillation*.tw.
4	atrium fibrillation*.tw.
5	exp Catheter Ablation/
6	atrial ablation*.tw.
7	(electric* adj2 ablation*).tw.
8	catheter ablation*.tw.
9	(radiofrequency adj2 ablation*).tw.
10	pulmonary vein isolation*.tw.
11	or/1-11
Intervention Keywords:	
12	exp Exercise/
13	exp Exercise Therapy/
14	exp Exercise Tolerance/
15	exp Physical Exertion/
16	exercis*.tw.
17	exp Physical Fitness/
18	exp "Physical Education and Training"/
19	(fitness or fitter or fit).tw.
20	(muscle* adj3 (train* or activ*)).tw.
21	(train* adj5 (strength* or aerobic* or exercise*)).tw.
22	((aerobic or resistance) adj3 (train* or activ*)).tw.
23	(physical* adj5 (fit* or train* or therap* or activ* or strength or endur* or exert* or capacit*)).tw.
24	((exercise* or fitness) adj3 (treat* or interven* or program* or train* or physical or activ*)).tw.
25	exp Exercise Tolerance/
26	(exercis* adj2 (toleran* or capacity)).tw.
27	exp Rehabilitation/
28	exp "Activities of Daily Living"/
29	exp Rehabilitation Centers/
30	rehabilitat*.tw.
31	kinesiotherap*.tw.
32	(("lifestyle" or life-style) adj5 activ$).tw.
33	(("lifestyle" or life-style) adj5 physical$).tw.
34	exp Patient Education as Topic/
35	(patient* adj5 educat*).tw.
36	((lifestyle or life-style) adj5 (interven* or program* or treatment*)).tw.
37	exp Self Care/
38	(self adj5 (manag* or care or motivate*)).tw.
39	exp Psychotherapy/
40	psychotherap*.tw.
41	(psycholog* adj5 intervent*).tw.
42	exp Counseling/
43	(counselling or counseling).tw.
44	((behavior* or behaviour*) adj5 (modify or modificat* or therap* or change)).tw.
45	(psycho-educat* or psychoeducat*).tw.
46	(motivat* adj5 (intervention or interv*)).tw.
47	exp Health Education/
48	(health adj5 educat*).tw.
49	(psychosocial or psycho-social).tw.
50	(cognitive adj2 behav*).tw.
51	or/12-50
52	11 and 51
MEDLINE (via Ovid) search strategy	
Participant Keywords:	
1	exp Atrial Fibrillation/
2	atrial fibrillation*.tw.
3	auricular fibrillation*.tw.
4	atrium fibrillation*.tw.
5	exp Catheter Ablation/
6	atrial ablation*.tw.
7	(electric* adj2 ablation*).tw.
8	catheter ablation*.tw.
9	(radiofrequency adj2 ablation*).tw.
10	pulmonary vein isolation*.tw.
11	or/1-10
Intervention Keywords:	
12	exp Exercise/
13	exp Exercise Therapy/
14	exp Exercise Tolerance/
15	exp Physical Exertion/
16	exercis*.tw.
17	exp Physical Fitness/
18	exp "Physical Education and Training"/
19	(fitness or fitter or fit).tw.
20	(muscle* adj3 (train* or activ*)).tw.
21	(train* adj5 (strength* or aerobic* or exercise*)).tw.
22	((aerobic or resistance) adj3 (train* or activ*)).tw.
23	(physical* adj5 (fit* or train* or therap* or activ* or strength or endur* or exert* or capacit*)).tw.
24	((exercise* or fitness) adj3 (treat* or interven* or program* or train* or physical or activ*)).tw.
25	exp Exercise Tolerance/
26	(exercis* adj2 (toleran* or capacity)).tw.
27	exp Rehabilitation/
28	exp "Activities of Daily Living"/
29	exp Rehabilitation Centers/
30	rehabilitat*.tw.
31	kinesiotherap*.tw.
32	(("lifestyle" or life-style) adj5 activ$).tw.
33	(("lifestyle" or life-style) adj5 physical$).tw.
34	exp Patient Education as Topic/
35	(patient* adj5 educat*).tw.
36	((lifestyle or life-style) adj5 (interven* or program* or treatment*)).tw.
37	exp Self Care/
38	(self adj5 (manag* or care or motivate*)).tw.
39	exp Psychotherapy/
40	psychotherap*.tw.
41	(psycholog* adj5 intervent*).tw.
42	exp Counseling/
43	(counselling or counseling).tw.
44	((behavior* or behaviour*) adj5 (modify or modificat* or therap* or change)).tw.
45	(psycho-educat* or psychoeducat*).tw.
46	(motivat* adj5 (intervention or interv*)).tw.
47	exp Health Education/
48	(health adj5 educat*).tw.
49	(psychosocial or psycho-social).tw.
50	(cognitive adj2 behav*).tw.
51	or/12-50
52	11 and 51
Study design Keywords:	
53	randomized controlled trial.pt.
54	controlled clinical trial.pt.
55	randomized.ab.
56	placebo.ab.
57	drug therapy.fs.
58	randomly.ab.
59	trial.ab.
60	groups.ab.
61	or/53-60
62	exp animals/ not humans.sh.
63	61 not 62
64	52 and 63
Embase (via ProQuest Health and Medical) search strategy	
Participant Keywords:	
1	MESH.EXACT.EXPLODE("Atrial Fibrillation") OR TI,AB("atrial fibrillation*") OR TI,AB("auricular fibrillation*") OR TI,AB("atrium fibrillation*") OR MESH.EXACT.EXPLODE("Catheter Ablation") OR TI,AB(" atrial ablation*") OR (TI,AB(electric*) NEAR/2 TI,AB(ablation*)) OR TI,AB("catheter ablation*") OR (TI,AB(radiofrequency) NEAR/2 TI,AB(ablation*)) OR TI,AB(" pulmonary vein isolation*")
Intervention Keywords:	
2	MESH.EXACT.EXPLODE(Exercise) OR MESH.EXACT.EXPLODE("Exercise Therapy") OR MESH.EXACT.EXPLODE("Exercise Tolerance") OR MESH.EXACT.EXPLODE("Physical Exertion") OR TI,AB(exercis*) OR MESH.EXACT.EXPLODE("Physical Fitness") OR MESH.EXACT.EXPLODE("Physical Education and Training") OR (TI,AB(fitness) OR TI,AB(fitter) OR TI,AB(fit)) " OR" (TI,AB(muscle*) NEAR/3 (TI,AB(train*) OR TI,AB(activ*))) OR (TI,AB(train*) NEAR/5 (TI,AB(strength*) OR TI,AB(aerobic*) OR TI,AB(exercise*))) " OR" ((TI,AB(aerobic) OR TI,AB(resistance)) NEAR/3 (TI,AB(train*) OR TI,AB(activ*))) OR (TI,AB(physical*) NEAR/5 (TI,AB(fit*) OR TI,AB(train*) OR TI,AB(therap*) OR TI,AB(activ*) OR TI,AB(strength) OR TI,AB(endur*) OR TI,AB(exert*) OR TI,AB(capacit*))) OR ((TI,AB(exercise*) OR TI,AB(fitness)) NEAR/3 (TI,AB(treat*) OR TI,AB(interven*) OR TI,AB(program*) OR TI,AB(train*) OR TI,AB(physical) OR TI,AB(activ*))) OR MESH.EXACT.EXPLODE("Exercise Tolerance") OR (TI,AB(exercis*) NEAR/2 (TI,AB(toleran*) OR TI,AB(capacity))) OR MESH.EXACT.EXPLODE(Rehabilitation) OR MESH.EXACT.EXPLODE("Activities of Daily Living") OR MESH.EXACT.EXPLODE("Rehabilitation Centers") OR TI,AB(rehabilitat*) OR TI,AB(kinesiotherap*) OR ((TI,AB(lifestyle) OR TI,AB(life-style)) NEAR/5 TI,AB(activ?)) OR ((TI,AB(lifestyle) OR TI,AB(life-style)) NEAR/5 TI,AB(physical?)) OR MESH.EXACT.EXPLODE("Patient Education as Topic") " OR" (TI,AB(patient*) NEAR/5 TI,AB(educat*)) OR ((TI,AB(lifestyle) OR TI,AB(life-style)) NEAR/5 (TI,AB(interven*) OR TI,AB(program*) OR TI,AB(treatment*))) OR MESH.EXACT.EXPLODE("Self Care") OR (TI,AB(self) NEAR/5 (TI,AB(manag*) OR TI,AB(care) OR TI,AB(motivate*))) OR MESH.EXACT.EXPLODE(Psychotherapy) OR TI,AB(psychotherap*) OR (TI,AB(psycholog*) NEAR/5 TI,AB(intervent*)) OR MESH.EXACT.EXPLODE(Counseling) OR (TI,AB(counselling) OR TI,AB(counseling)) OR ((TI,AB(behavior*) OR TI,AB(behaviour*)) NEAR/5 (TI,AB(modify) OR TI,AB(modificat*) OR TI,AB(therap*) OR TI,AB(change))) OR (TI,AB(psycho-educat*) OR TI,AB(psychoeducat*)) OR (TI,AB(motivat*) NEAR/5 (TI,AB(intervention) OR TI,AB(interv*))) OR MESH.EXACT.EXPLODE("Health Education") OR (TI,AB(health) NEAR/5 TI,AB(educat*)) OR (TI,AB(psychosocial) OR TI,AB(psycho-social)) OR (TI,AB(cognitive) NEAR/2 TI,AB(behav*))
Study Keywords:	
3	TI,AB("randomized controlled trial") OR TI,AB("controlled clinical trial") OR TI,AB(randomized) OR TI,AB(placebo) OR TI,AB("drug therapy") OR TI,AB(randomly) OR TI,AB(trial) OR TI,AB(groups)
4	#1 AND #2 AND #3
CINAHL (via EBSCOhost) search strategy	
Participant Keywords:	
1	((MH "Atrial Fibrillation"+) ) OR ((TI "atrial fibrillation*" OR AB "atrial fibrillation*")) OR ((TI "auricular fibrillation*" OR AB "auricular fibrillation*")) OR ((TI "atrium fibrillation*" OR AB "atrium fibrillation*")) OR ((MH "Catheter Ablation"+) ) OR ((TI "atrial ablation*" OR AB "atrial ablation*")) OR (((TI electric* OR AB electric*) N2 (TI ablation* OR AB ablation*)) ) OR ((TI "catheter ablation*" OR AB "catheter ablation*")) OR (((TI radiofrequency OR AB radiofrequency) N2 (TI ablation* OR AB ablation*)) ) OR ((TI "pulmonary vein isolation*" OR AB "pulmonary vein isolation*"))
Intervention Keywords:	
2	((MH Exercise+) ) OR ((MH "Exercise Therapy"+) ) OR ((MH "Exercise Tolerance"+) ) OR ((MH "Physical Exertion"+) ) OR ((TI exercis* OR AB exercis*)) OR ((MH “Physical Fitness”+) ) OR ((MH “Physical Education and Training”+)) OR (((TI fitness OR AB fitness) OR (TI fitter OR AB fitter) OR (TI fit OR AB fit)) ) OR (((TI muscle* OR AB muscle*) N3 ((TI train* OR AB train*) OR (TI activ* OR AB activ*))) ) OR (((TI train* OR AB train*) N5 ((TI strength* OR AB strength*) OR (TI aerobic* OR AB aerobic*) OR (TI exercise* OR AB exercise*))) ) OR ((((TI aerobic OR AB aerobic) OR (TI resistance OR AB resistance)) N3 ((TI train* OR AB train*) OR (TI activ* OR AB activ*))) ) OR (((TI physical* OR AB physical*) N5 ((TI fit* OR AB fit*) OR (TI train* OR AB train*) OR (TI therap* OR AB therap*) OR (TI activ* OR AB activ*) OR (TI strength OR AB strength) OR (TI endur* OR AB endur*) OR (TI exert* OR AB exert*) OR (TI capacit* OR AB capacit*)))) OR ((((TI exercise* OR AB exercise*) OR (TI fitness OR AB fitness)) N3 ((TI treat* OR AB treat*) OR (TI interven* OR AB interven*) OR (TI program* OR AB program*) OR (TI train* OR AB train*) OR (TI physical OR AB physical) OR (TI activ* OR AB activ*))) ) OR ((MH "Exercise Tolerance"+) ) OR (((TI exercis* OR AB exercis*) N2 ((TI toleran* OR AB toleran*) OR (TI capacity OR AB capacity)))) OR ((MH Rehabilitation+)) OR ((MH "Activities of Daily Living"+)) OR ((MH "Rehabilitation Centers"+) ) OR ((TI rehabilitat* OR AB rehabilitat*)) OR ((TI kinesiotherap* OR AB kinesiotherap*)) OR ((((TI lifestyle OR AB lifestyle) OR (TI life-style OR AB life-style)) N5 (TI activ? OR AB activ?))) OR ((((TI lifestyle OR AB lifestyle) OR (TI life-style OR AB life-style)) N5 (TI physical? OR AB physical?))) OR ((MH "Patient Education as Topic"+) ) OR (((TI patient* OR AB patient*) N5 (TI educat* OR AB educat*))) OR ((((TI lifestyle OR AB lifestyle) OR (TI life-style OR AB life-style)) N5 ((TI interven* OR AB interven*) OR (TI program* OR AB program*) OR (TI treatment* OR AB treatment*)))) OR ((MH "Self Care"+)) OR (((TI self OR AB self) N5 ((TI manag* OR AB manag*) OR (TI care OR AB care) OR (TI motivate* OR AB motivate*)))) OR ((MH Psychotherapy+)) OR ((TI psychotherap* OR AB psychotherap*)) OR (((TI psycholog* OR AB psycholog*) N5 (TI intervent* OR AB intervent*))) OR ((MH Counseling+)) OR (((TI counselling OR AB counselling) OR (TI counseling OR AB counseling))) OR ((((TI behavior* OR AB behavior*) OR (TI behaviour* OR AB behaviour*)) N5 ((TI modify OR AB modify) OR (TI modificat* OR AB modificat*) OR (TI therap* OR AB therap*) OR (TI change OR AB change)))) OR (((TI psycho-educat* OR AB psycho-educat*) OR (TI psychoeducat* OR AB psychoeducat*))) OR (((TI motivat* OR AB motivat*) N5 ((TI intervention OR AB intervention) OR (TI interv* OR AB interv*)))) OR ((MH "Health Education"+) ) OR (((TI health OR AB health) N5 (TI educat* OR AB educat*))) OR (((TI psychosocial OR AB psychosocial) OR (TI psycho-social OR AB psycho-social))) OR (((TI cognitive OR AB cognitive) N2 (TI behav* OR AB behav*)))
Study Design Keywords:	
3	((PT "randomized controlled trial") OR (PT "controlled clinical trial") OR (AB randomized) OR (AB placebo) OR ("Drug Therapy") OR (AB randomly) OR (AB trial) OR (AB groups) OR (MH “Crossover Design”)) NOT ((MH animals+) NOT (MH humans))
4	#1 AND #2 AND #3
Web of Science search strategy	
Participant Keywords:	
1	TS=((atrial fibrillation*) OR (auricular fibrillation*) OR (atrium fibrillation*)) OR TS=((atrial ablation*) OR (electric* near/2 ablation*) OR (catheter ablation*) OR (radiofrequency near/2 ablation*)OR (pulmonary vein isolation*))
Intervention Keywords:	
2	TS=((exercis*) OR (fitness or fitter or fit) OR (muscle* near/3 (train* or activ*)) OR (train* near/5 (strength* or aerobic* or exercise*)) OR ((aerobic or resistance) near/3 (train* or activ*)) OR (physical* near/5 (fit* or train* or therap* or activ* or strength or endur* or exert* or capacit*)) OR ((exercise* or fitness) near/3 (treat* or interven* or program* or train* or physical or activ*)) OR (exercis* near/2 (toleran* or capacity)) OR (rehabilitat*) OR (kinesiotherap*) OR (("lifestyle" or life-style) near/5 activ*) OR (("lifestyle" or life-style) near/5 physical*) OR (patient* near/5 educat*) OR ((lifestyle or life-style) near/5 (interven* or program* or treatment*)) OR (self near/5 (manag* or care or motivate*)) OR (psychotherap*) OR (psycholog* near/5 intervent*) OR (counselling or counseling) OR ((behavior* or behaviour*) near/5 (modify or modificat* or therap* or change)) OR (psycho-educat* or psychoeducat*) OR (motivat* near/5 (intervention or interv*)) OR (health near/5 educat*) OR (psychosocial or psycho-social) OR (cognitive near/2 behav*))
Study Design Keywords:	
3	TS=((random* or blind* or allocat* or assign* or trial* or placebo* or crossover* or cross-over* ))
4	#1 AND #2 AND #3
PEDro search strategy	
1	Advance search
2	Abstract & Title: atrial fibrillation
3	Method: clinical trial
ClinicalTrials.gov search strategy	
1	Condition or disease: Atrial Fibrillation
2	Intervention/Treatment: Rehabilitation OR Exercise
3	Study Type: Interventional

**Table 5:** List of excluded studies

Wrong population		
Author	Title	Year
Pogosova et al.	Different preventive counseling programs for patients with paroxysmal atrial fibrillation after catheter ablation	2017
Seo et al.	The effect of cardiac rehabilitation at 4 weeks postoperatively on quality of life in patients treated with totally thoracoscopic ablation.	2019
Seo et al.	Can exercise-based cardiac rehabilitation increase physical activity in patients who have undergone total thoracoscopic ablation?	2021
Elliott et al.	Impact of sex on outcomes following aerobic exercise training in patients with symptomatic atrial fibrillation	2022
Wrong comparison		
Bao et al.	Improvement of sleep quality by home-based exercise rehabilitation in telehealth mode in patients with atrial fibrillation after catheter ablation. Journal of arrhythmia.	2019
Non-RCT		
Mohanty et al.	Aggressive life-style modifications facilitate arrhythmia-free survival in atrial fibrillation patients with coexistent metabolic syndrome and sleep apnea having sporadic recurrence following first ablation procedure	2013
Tang et al.	Self-rating level of perceived exertion for guiding exercise intensity during a 12-week cardiac rehabilitation programme and the influence of heart rate reducing medication	2016
Tang et al.	Patient's preference for exercise setting and its influence on the health benefits gained from exercise-based cardiac rehabilitation	2017
Aoyama et al.	Cardiac rehabilitation after catheter ablation of atrial fibrillation in patients with left ventricular dysfunction.	2021
Same studies		
Risom et al.	A randomized clinical trial investigating the effect and meaning of integrated rehabilitation versus usual follow-up of patients treated for atrial fibrillation with radio frequency ablation	2012
Risom et al.	Copenheart.rfa a randomized clinical trail of integrated rehabilitation for patients treated for atrial fibrillation with radiofrequency ablation	2013
Risom et al	The effect of integrated cardiac rehabilitation versus treatment as usual for atrial fibrillation patients treated with ablation: the randomised CopenHeart _RFA_ trial protocol.	2015
Risom et al.	Comprehensive rehabilitation for patients treated for atrial fibrillation with ablation; Results from the CopenHeartRFA randomized trial.	2015
Wagner et al.	Gender differences in effect of cardiac rehabilitation among patients with atrial fibrillation treated with radiofrequency ablation results from the Copenheart RFA trial	2016
Risom et al.	Randomised clinical trial of comprehensive rehabilitation for patients treated for atrial fibrillation with ablation: long-term impact on quality of life and physical capacity.	2017
Risom et al.	Exploring why different effects of a psychoeducational intervention were found in a rehabilitation programme for patients treated with ablation for atrial fibrillation. a mixed methods study	2017
Makhinova et al.	The effects of physical rehabilitation program on physical activity and inflammatory markers in patients with atrial fibrillation after ablation	2017
Wagner et al.	Sex differences in health status and rehabilitation outcomes in patients with atrial fibrillation treated with ablation: Results from the CopenHeartRFA trial.	2018
Risom et al.	Effect of rehabilitation on sleep quality after ablation for atrial fibrillation: Data from a randomized trial	2018
Risom et al.	Exploring the mechanism of effectiveness of a psychoeducational intervention in a rehabilitation program (CopenHeartRFA) for patients treated with ablation for atrial fibrillation: A mixed methods study	2019
Risom et al.	Cardiac rehabilitation for patients treated for atrial fibrillation with ablation has long-term effects: 12-and 24-month follow-up results from the randomized CopenHeartRFA trial.	2020
NCT04414007	The application of internet+ home-based cardiac rehabilitation in atrial fibrillation patients after RFCA	2020
Pogosova et al.	Efficacy of secondary prevention and rehabilitation programs with distant support in patients with atrial fibrillation after intervention procedures: impact on psychological status	2022
Cai et al.	A novel model of home-based, patient-tailored and mobile application-guided cardiac telerehabilitation in patients with atrial fibrillation: A randomised controlled trial.	2022
Ongoing study		
Kristýna et al.	Effect of complex weight-reducing interventions on rhythm control in obese subjects with atrial fibrillation	2020
NCT05450731	Effect of cardiac rehabilitation exercise on the recurrence of atrial fibrillation in post-ablation patients: a Randomized Controlled Pilot Study	2022
ACTRN12623000456651	Randomised controlled trial of exercise training versus standard medical care on atrial fibrillation recurrence after ablation in patients with symptomatic atrial fibrillation	2023
